# CArG-driven GADD45α activated by resveratrol inhibits lung cancer cells

**DOI:** 10.18632/genesandcancer.62

**Published:** 2015-05

**Authors:** Qiwen Shi, Werner Geldenhuys, Vijaykumar Sutariya, Anupam Bishayee, Isha Patel, Deepak Bhatia

**Affiliations:** ^1^ Department of Pharmaceutical Sciences, Northeast Ohio Medical University, Rootstown, Ohio, USA; ^2^ School of Biomedical Sciences, Kent State University, Kent, Ohio, USA; ^3^ Department of Pharmaceutical Sciences, University of South Florida, Tampa, Florida, USA; ^4^ Department of Pharmaceutical Sciences, Larkin Health Sciences Institute College of Pharmacy, Miami, Florida, USA; ^5^ Bernard J. Dunn School of Pharmacy, Shenandoah University, Ashburn, Virginia, USA

**Keywords:** resveratrol, CArG elements, Egr-1, GADD45a, gene therapy, synthetic promoters

## Abstract

We report anticarcinogenic effects of suicide gene therapy that relies on the use of resveratrol-responsive CArG elements from the Egr-1 promoter to induce GADD45α. In A549 lung cancer cells, endogenous GADD45α was not induced upon resveratrol treatment. Therefore, induction of exogenous GADD45α resulted in growth inhibition. Resveratrol transiently induced Egr-1 through ERK/JNK-ElK-1. Hence, we cloned natural or synthetic Egr-1 promoter upstream of GADD45α cDNA to create a suicide gene therapy vector. Since natural promoter may have antagonized effects, we tested synthetic promoter that contains either five, six or nine repeats of CArG elements essential in the Egr-1 promoter to drive the expression of GADD45α upon resveratrol treatment. Further analysis confirmed that both synthetic promoter and natural Egr-1 promoter were able to “turn on” the expression of GADD45α when combined with resveratrol, and subsequently led to suppression of cell proliferation and apoptosis.

## INTRODUCTION

Lung cancer is the leading cause of cancer-related death in both men and women around the world. Approximately 85% of all lung cancers are non-small cell lung carcinoma (NSCLC), with lung adenocarcinoma being the most common histologic type in the United States. Most patients with advanced stage disease are not candidates for surgical resection but are subjected to platinum based chemotherapy and radiation therapy alone or in combination. Unfortunately, despite these therapies, prognosis is still poor with an overall 5-year survival rate of only 15% [2].

Growth arrest and DNA damage inducible 45α (GADD45α) is a stress-inducible gene transcriptionally activated by growth-arrest-associated proteins including p53, FOXO3a, ATF4 and C/EBP, and repressed by growth stimulating factors such as c-Myc and AKT [3]. The main function of GADD45α in the cell cycle is to arrest G2/M transition through disrupting Cdk1/Cyclin B1 interaction after DNA damage [5], and interestingly, GADD45α-mediated G2/M arrest is dependent on normal p53 function [1]. Other possible roles of GADD45α include activating p38/JNK pathway which is associated with apoptosis [6], regulating Aurora-A and Nek2 which have functions in the maintenance of genomic stability [3], disrupting elongation factor 1α (EF-1α)-mediated microtubule bundling to induce apoptosis [12], and suppressing mTOR/STAT3 pathway to restrain angiogenesis [7]. Previous studies have demonstrated that inactivation of GADD45α may associate with the development of malignancy as GADD45α-null mice and p53-deficient mice have similar phenotypes such as increased radiation carcinogenesis, a low frequency of exencephaly and genomic instabilities which are exemplified by aneuploidy, chromosomal aberrations, gene amplification, centrosome amplification, abnormal mitosis and cytokinesis [35]. Besides, GADD45α-null mice are susceptible to DNA damage-induced tumors [36]. Downregulation and aberrant expression of GADD45α have been established in many types of cancer. Higashi et al. showed that GADD45α mRNA level was 90% lower in NSCLC tumors than that in histological normal lung tissues [8]. Al-Romaih et al. reported GADD45α was epigenetically inactivated in various types of tumor cell lines such as osteosarcoma cells [9]. Importantly, the fact that reactivation of GADD45α results in the induction of cancer cell apoptosis validates the feasibility of GADD45α-targeted cancer therapy [6, 10-12]. A recent report showed an increased suppression in pancreatic cancer cell growth through inducing apoptosis and cell cycle arrest after cells were transfected with adenoviral construct of GADD45α [37].

Early growth response 1 (Egr-1) is a transcription factor rapidly and transiently induced by a broad variety of stimuli such as growth factors [13], differentiation signals [14], ionizing radiation (IR) [15] and shear stress [16]. It directly binds to GADD45α promoter and activates the transcription of GADD45α upon UVB exposure [32] or arsenic insult [17]. The activation of Egr-1 is p53-independent and the radiation and chemotherapy-inducible regions of Egr-1 promoter have been identified as 10-nucleotide motifs of the consensus sequence CC(A/T)6GG which are also known as CArG elements [18-19]. The CArG elements are often referred to as serum response elements (SREs) and involved in the regulation of multiple immediate-early genes after mitogenic stimulation [26]. The precise mechanism of radiation- and chemotherapy-responsive property of CArG elements has yet to be elucidated. Reactive oxygen species (ROS) formation through radiation and chemotherapy is one explanation, and complexes of phosphorylated transcription factors and accessory proteins are known to promote gene transcription by binding to CArG motifs [20]. In addition, only the CArG elements in the 5′ distal enhancer region of Egr-1 gene appear to be responsive to radiation and chemotherapeutic agents [21]. A 425-nucleotide region of murine Egr-1 promoter was sufficient to drive the expression of tumor necrosis factor α (TNFα) beyond activation when being inserted upstream of TNFα cDNA [22]. Furthermore, Scott et al. found that increasing the number of CArG elements and altering the core A/T sequence in these motifs enhanced promoter radiation-responsiveness [20].

Resveratrol is a phytochemical found in several dietary sources, and it is a leading candidate in the fight against age-related disorders. Resveratrol affects the activity of transcriptional factors involved in proliferation and stress responses such as NF-kB, AP1 and Egr-1 [24], and targets signaling pathways regulating cell proliferation, growth and apoptosis, inflammation, and tumor invasion, angiogenesis and metastasis [23]. Resveratrol is well tolerated and relatively safe [38], so has been considered as an extended candidate in TNFerade study to benefit patients who cannot tolerate even low doses of radiation or chemotherapy and for long-term gene therapy application [25]. TNFerade is a replication-deficient adenovector carrying CArG elements to regulate the expression of TNFα, a pro-apoptotic cytokine. TNFerade has been effective in combination with radiotherapy or chemotherapy in preclinical studies, leading to direct tumor killing, anti-angiogenesis and synergistic interaction with radiation or chemotherapeutic agents, and has passed phase I/II clinical trials for pancreatic cancer [41].

The overall goal of our study is to establish a novel treatment paradigm for NSCLC by creating a suicide gene therapy vector that expresses GADD45α protein under the control of resveratrol-activated CArG elements. Here, we determined the MAPKs involved in resveratrol-induced Egr-1 expression and assessed the therapeutic efficacy of our suicide gene therapy strategy.

## RESULTS

### Induction of Egr-1 expression by resveratrol in lung cancer cells

**Figure 1:**
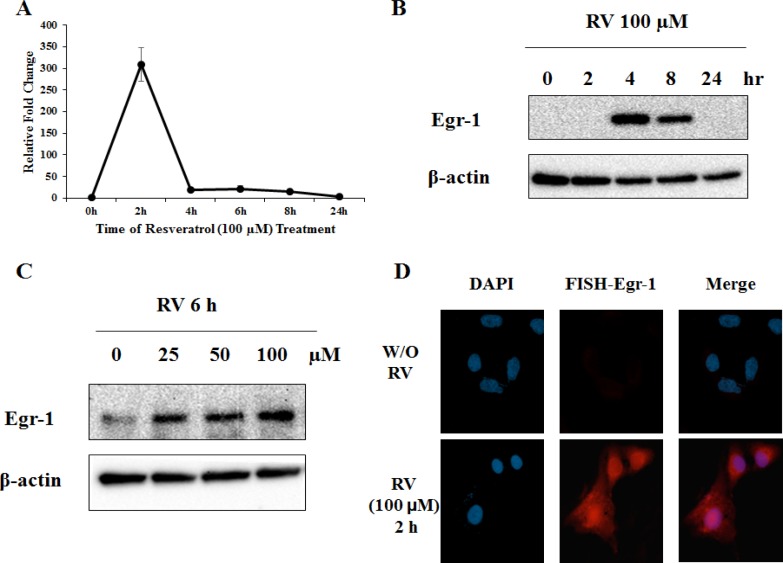
Resveratrol induces Egr-1 expression **A**. A549 cells were treated with 100 μM of resveratrol for indicated hours and Egr-1 mRNA levels were measured by real-time RT-PCR. The Egr-1 mRNA expression was adjusted to GAPDH (housekeeping gene) and normalized to untreated control. **B.** and **C**. Egr-1 and β-actin (loading control) protein expression was determined by western blotting after indicated resveratrol treatment. **D**. Fluorescent *In Situ* Hybridization assay was used to detect Egr-1 mRNA level in non-treated and resveratrol-treated cells. Images were taken on Axio Imager M2 at 400X magnification.

Resveratrol is able to activate Egr-1 expression in several cancers including pancreatic [25] and colorectal cancer [27]. To determine whether resveratrol is an effective inducer of Egr-1 expression in lung cancer, A549 cells were incubated with 100 μM of resveratrol, and mRNA level and protein expression were measured at different time points by real-time RT-PCR and western blotting respectively. The induction of Egr-1 mRNA and protein reached maximum 2 h and 4 h respectively after resveratrol treatment (Figure 1A and 1B). In addition, the expression of Egr-1 protein was measured after cells were treated with 0, 25, 50 and 100 μM of resveratrol for 6 h, and a dose-dependent induction was detected (Figure 1C). Fluorescence in situ hybridization (FISH) assay reinforced that Egr-1 mRNA level was significantly induced 2 h after resveratrol exposure (Figure 1D). These results suggest that resveratrol is able to induce Egr-1 expression rapidly and transiently.

### Characterization of Elk-1 expression following resveratrol treatment

**Figure 2:**
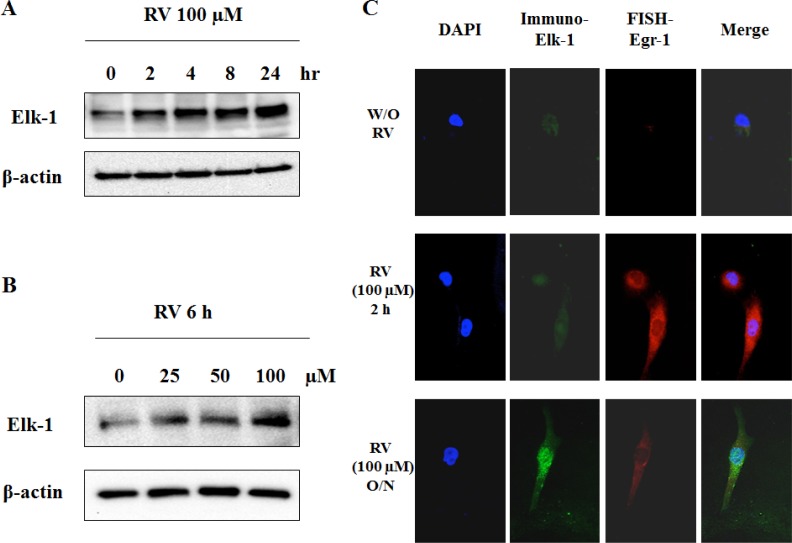
Elk-1 is activated by resveratrol **A**. and **B**. Western blotting was used to detect Egr-1 protein expression in cells treated with different concentrations of resveratrol for indicated hours. **C**. Fluorescent *In Situ* Hybridization staining of Egr-1 and immunofluorescence staining of Elk-1 in resveratrol-treated cells and control cells. Images were taken on Axio Imager M2 at 400X magnification.

Elk-1 is a major downstream of the MAPK pathway and its activation leads to increased DNA binding, ternary complex formation and SRE-mediated transcription [28]. As shown previously, resveratrol induced apoptosis in both thyroid cancer cells and JB6 mouse epidermal cells in a MAPK-dependent manner [29, 30]. To determine whether Elk-1 is a participator in resveratrol-activated signaling cascade, Elk-1 protein levels were determined after cells were treated with indicated concentrations of resveratrol for indicated hours. The results showed that Elk-1 protein expression was increased by resveratrol in a time- and dose-dependent manner (Figure 2A and 2B). The resveratrol-induced Elk-1 expression was also confirmed by immunofluorescence staining in which the expression of Elk-1 protein was significantly augmented by overnight treatment with resveratrol (100 μM) (Figure 2C). Taken together, Elk-1 is confirmed as a target of resveratrol in lung cancer.

### MAPK and Elk-1 for resveratrol-induced Egr-1 biosynthesis

**Figure 3:**
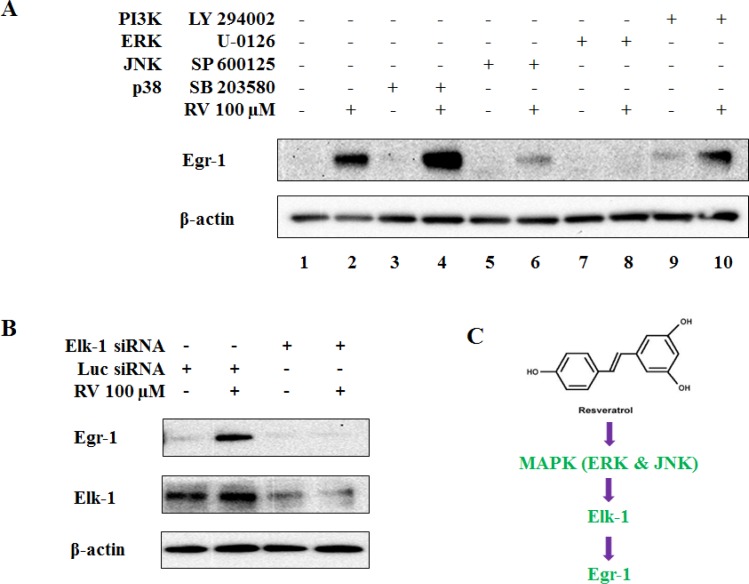
MAPK pathway is involved in resveratrol-induced Egr-1 expression **A**. Cells were treated with or without 100 μM of resveratrol for 6 h after pretreatment with indicated inhibitors for 1 h, and Egr-1 protein level were examined by western blotting. **B**. Cells were exposed to 100 μM of resveratrol for 6 h after transfected with either Elk-1 siRNA or Luciferase siRNA. **C**. The summary of ERK/JNK-Elk-1-Egr-1 pathway: resveratrol sequentially activates MAPK cascade, Elk-1 and Egr-1.

Activation of individual MAPK by a certain stimulus depends on the cell line, time and dose applied. Therefore, it is important to identify the involvement of each MAPK in resveratrol-mediated Egr-1 induction in A549 cells. We also tested the role of PI3K/AKT pathway as it is an important pathway to control multiple cell functions including cell proliferation, growth and survival. Cells were pretreated with p38 inhibitor (SB 203580, 10 μM), JNK inhibitor (SP 600125, 20 μM), ERK inhibitor (U-0126, 10 μM) and PI3K/AKT inhibitor (LY 294002, 10 μM) respectively for 1 h and then exposed to either 0.1% DMSO or 100 μM of resveratrol for additional 6 h. The analysis of Egr-1 protein levels indicated that SP 600125 and U-0126 were able to block resveratrol-mediated Egr-1 induction (Figure 3A, lane 6 and 8), whereas SB 203580 and LY 294002 had no significant effects on resveratrol-induced Egr-1 protein expression (Figure 3A, lane 4 and 10). Given that Elk-1 is a well-described downstream target of MAPK pathways as well as a potential regulator of Egr-1 promoter [31], we investigated whether resveratrol-induced Egr-1 expression relies on Elk-1 activity. The results demonstrated that the silencing of Elk-1 by siRNA transfection abolished the induction of Egr-1 protein by resveratrol (Figure 3B). All the data demonstrate that the normal function of JNK, ERK and Elk-1 is necessary for resveratrol-activated Egr-1 expression, and indicate that JNK/ERK-Elk-1-Egr-1 is a signaling pathway triggered by resveratrol (Figure 3C).

### Marginal effects of resveratrol on GADD45α expression in lung cancer

**Figure 4:**
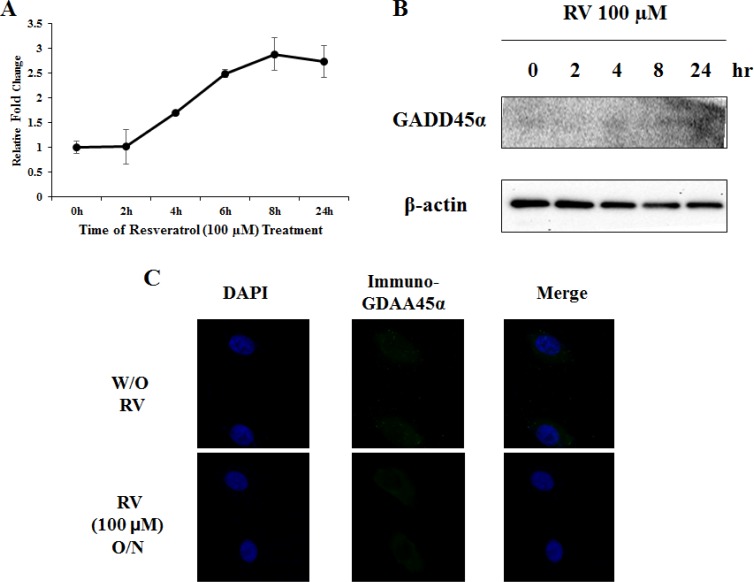
Resveratrol is not sufficient to activate GADD45α **A**. and **B**. GADD45α protein expression and mRNA level were measured by western blotting and real-time RT PCR after cells were treated with 100 μM of resveratrol for various hours. **C**. Cells were treated with or without resveratrol overnight and performed anti-GADD45α immunofluorescent staining. Images were taken on Axio Imager M2 at 400X magnification.

As we previously reported, Egr-1 directly binds to GADD45α promoter and activates GADD45α transcription in the presence of arsenic in normal lung cells [17]. To determine whether resveratrol is able to induce GADD45α in lung cancer, A549 cells were treated with 100 μM of resveratrol up to 24 h and both mRNA level and protein expression were measured. GADD45α mRNA was slightly increased in a time-dependent manner (Figure 4A) but GADD45α protein was not detected by western blotting (Figure 4B). Moreover, in immunofluorescence staining, no significant change was observed regarding GADD45α protein expression between cells treated with 100 μM of resveratrol overnight and non-treated cells (Figure 4C). In conclusion, our data suggest that resveratrol has limited effects on the induction of GADD45α expression.

### Optimization of resveratrol-responsive synthetic Egr-1 promoters

**Figure 5:**
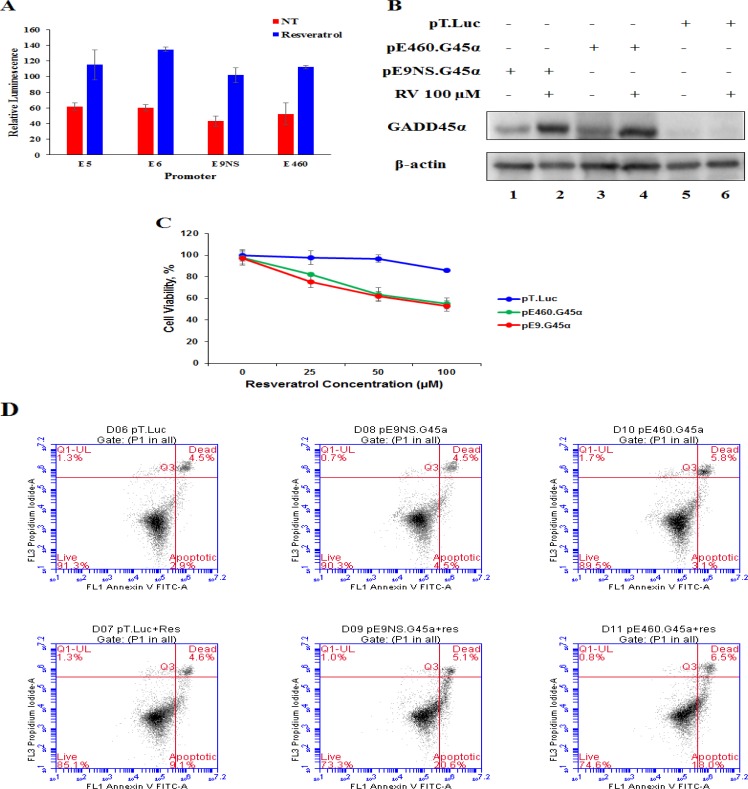
Both synthetic and natural promoters are able to overexpress GADD45α to induce cell apoptosis when combined with resveratrol **A**. Cells were co-transfected with luciferase constructs containing either the synthetic CArG promoter (pE5, pE6 and pE9NS) or the natural Egr-1 promoter (pE460) and Renilla luciferase control reporter vector (pRL-TK) followed by resveratrol or non-treatment. Relative luminescence was plotted as the average of triplicate experiment and represented as mean ± SE. **B**. Level of GADD45α protein was detected by western blotting after cells were transfected with either suicide gene therapy vector (pE460.G45α or pE9NS.G45α) or control vector (pT.Luc) and then treated with or without 100 μM of resveratrol for 24 h. **C**. MTT assay was performed for cells pre-transfected with pE460.G45α, pE9NS.G45α or pT.Luc and treated with different doses of resveratrol for 24 h. **D**. Cells were exposed to 100 μM of resveratrol for 24 h after transfected with control or suicide gene therapy constructs. Apoptosis was determined by FACS of Annexin V-FITC and PI stained cells.

In order to confirm the potential of combining CArG-mediated gene therapy with resveratrol, we tested the resveratrol-responsiveness of synthetic promoters E5 (five repeats of prototype CArG sequence (CCTTATTTGG), E6 (six repeats of prototype CArG sequence) and E9NS (nine repeats of new CArG sequence (CCATATAAGG), and the natural Egr-1 promoter E460 (460 nucleotides upstream of transcription start site). The relative luminescence was monitored by dual-luciferase assay reporter system. All synthetic promoters (E5, E6 and E9NS) were activated 1.75- to 2.25-fold by resveratrol (Figure 5A). Therefore, due to lack of statistical difference in resveratrol-responsiveness amongst these synthetic promoters, further studies only included E9NS promoter [20].

### Therapeutic effects of suicide gene therapy

To confirm the overexpression of GADD45α protein through the combination of our gene therapy vector and resveratrol treatment delays lung cancer progression and increases cancer cell apoptosis, we transfected A549 cells with pT.Luc (control vector), pE9NS.G45α or pE460.G45α for 24 h and then treated with either 0.1% DMSO or 100 μM of resveratrol for additional 24 h before proceeding to western blotting, MTT assay and Annexin-V/propidium iodide (PI) flow cytometric analysis. Immunoblotting data depicted that both pE9NS.G45α and pE460.G45α were able to drive the expression of GADD45α in A549 cells when activated by resveratrol (Figure 5B). The data from MTT assay and flow cytometric analysis suggested resveratrol alone had marginal suppression in cell growth and negligible induction in apoptosis, but resveratrol plus gene therapy vector (pE9NS.G45α or pE460.G45α) exhibited less than 50% cell viability and approximately 20% higher apoptosis as opposed to control (Figure 5C and 5D). Transfection with gene therapy vector but without resveratrol treatment did not significantly affect cell growth or apoptosis in these cells (Figure 5C and 5D).

## DISCUSSION

Our study demonstrated that resveratrol activated Egr-1 biosynthesis via ERK/JNK-Elk-1 signaling pathway in A549 cells. Similarly, Egr-1 has been reported to be induced by resveratrol through ERK but not p38 pathway in both normal and neoplastic human cells, and this induction is irrelevant to cellular p53 status [33]. In erythroleukemic cells, Egr-1 upregulation by resveratrol requires ERK1/2 activation and contributes to the chemopreventive and antiproliferative properties of resveratrol through downstream target p21Cip1 [39]. In general, instead of causing direct DNA damage, resveratrol exhibits its effects by interfering with DNA replication and cell division. Sirtuin 1 (SIRT1) is the major protein activated by resveratrol, but Bickenbach et al. found that SIRT1 inhibitor did not block resveratrol-induced Egr-1 expression [25], suggesting that resveratrol upregulates Egr-1 expression probably through other mechanisms.

The selection of GADD45α as the suicide gene is based on the assumptions that (i) dysregulation of GADD45α could be expected in lung cancer; (ii) a correct sequence of GADD45α can provide therapeutic measures; (iii) targeting the downstream pathway of signaling network is the most preferred over an upstream pathway that may have many biological processes compromised. In promyelocytic leukemia (HL-60) cells, resveratrol induces apoptosis through GADD45α upregulation [40]. On the contrary, in our study the mRNA level of GADD45α was increased by about 3 folds 24 h after resveratrol treatment, but no change in protein expression was detected either by immunofluorescence staining or western blotting. Furthermore, opposed to the effects in leukemia, resveratrol only showed weak cytotoxic effects and growth inhibition in lung cancer cells, indicating that resveratrol alone may not be effective in lung cancer treatment, but may facilitate other treatment strategies.

Although GADD45α fails to arrest cell cycle progression at G2/M checkpoint in p53-deficient cancer cells, it is still able to suppress cell proliferation via inducing apoptosis [43], indicating that GADD45α-mediated G2/M arrest depends on normal p53 function whereas GADD45α-induced apoptosis is irrelevant to p53. MTK1/MEKK4-p38/JNK pathway has been established as the mechanism through which GADD45α regulates apoptosis in response to environmental stresses [6], but the involvement of JNK activation remains controversial since GADD45α was not necessary in stress-induced JNK activation [34] and JNK is only marginally activated even after significant induction of GADD45α in HeLa cells [12]. Recently, Tong et al. posted a novel pathway elucidating the underlying mechanism of apoptosis mediated by stress-induced-GADD45α: GADD45α interacts with EF-1α, causing cytoskeleton destabilization which induces the release of Bim and its translocation to mitochondria, and the successive steps include the release of cytochrome c, the activation of caspase-9 and caspase-3, and ultimately apoptosis [12]. In our report, ectopic expression of GADD45α successfully suppressed cell proliferation and induced apoptosis in lung cancer. Additional investigations are required to determine whether the mechanism is G2/M arrest, destabilized cytoskeleton or enhancement of other apoptosis/growth arrest-related signaling pathways activated by resveratrol.

A safety switch, which is inactive but inducible when combined with low doses of standard regiments (platinum based therapy and/or radiation therapy) or natural compounds, could minimize toxicity in normal tissue and facilitate the controllable applications of gene therapy. The Egr-1 promoter has been widely studied for the regulation of therapeutic genes in the experimental models of radiation- and chemotherapy-mediated gene therapy, such as Ad.Egr-TNF11D [41] and the EGR1-CYP4B1/4-IM system [42]. However, besides the CArG elements, Egr-1 promoter contains other regulatory sites including two Sp1 binding sites, a cyclic adenosine monophosphate (cAMP) response element (CRE), a NF-κB binding site and an Egr binding sequence (EBS) [26]. The promoter E460 used in our study only contains 460 nucleotides upstream of Egr-1 transcription start site, but still includes binding sites for Sp1 and cAMP. Synthetic alternatives consisting of repeated CArG elements have been developed in order to eliminate potentially antagonistic binding sites. Scott et al. have optimized the synthetic promoter radiation-responsiveness based on the numbers, core sequences, spacing and context [20]. Among a series of synthetic promoters, the one carrying nine repeats of new sequence CArG elements but no extra nucleotide “spacer” sequence nor Sp1 binding site has obtained the greatest sensitivity to radiation [20]. The later study has demonstrated that the new sequence CArG element is more sensitive to neutron/X-ray radiation and chemotherapeutic agents than the prototype CArG sequence, and the activated synthetic promoter is sufficient to express downstream HSVtk [4]. In the present study, we have constructed a natural promoter E460 and three synthetic promoters E5, E6 and E9NS, and compared their responses to resveratrol. All promoters were responsive to resveratrol and no significant difference was observed between synthetic and natural promoters in term of resveratrol-responsiveness as well as therapeutic efficacy when associated with our gene therapy.

In conclusion, the present study confirmed the potential of synthetic promoters based on isolated CArG elements to selectively control therapeutic gene expression in the tumor and the feasibility of GADD45α to work as a therapeutic drug. Currently, our gene therapy is associated with resveratrol that is nontoxic and protective to normal cells but may have low bioavailability due to rapid metabolism. In future, this gene therapy vector may work synergistically with traditional lung cancer treatment such as radiation therapy and chemotherapy, and achieve better therapeutic effects even with low doses of radiotherapy and chemotherapy and simultaneously reduce the damage to normal tissues. Moreover, since GADD45α-induced apoptosis is not p53 dependent, our gene therapy may benefit patients regardless of p53 status in their tumors. Further investigation will be conducted with low doses of radiation and multiple chemotherapeutic agents in cell lines with various p53 status (Shi et al., Manuscript in preparation).

## MATERIALS AND METHODS

### Cell culture and media

A549, a human lung adenocarcinoma epithelial cell line, was obtained from the American Type Culture Collection (Manassas, VA). A549 was maintained in Dulbecco's modified Eagle's medium (DMEM) (Invitrogen Life Technologies, CA) supplemented with fetal bovine serum (10% v/v, Invitrogen/Gibco BRL, CA), penicillin (100 IU/ml) and streptomycin (100 μg/ml, Sigma, MO) at 37 °C with 5% CO2. All the cells were passaged when confluency was reached, usually every 2-4 days.

### Western blot analysis

After transfection or/and resveratrol treatment, the cells were washed with PBS and then harvested in RIPA buffer (Teknova, CA) containing 1X phosphatase inhibitor cocktail I and 1X protease inhibitor cocktail II (Boston BioProducts, MA). The lysis was sonicated with Branson 450 analog sonifier for 10 rounds at 20% duty cycle, 2-output control. Total protein (20-50 μg) for each sample was separated by electrophoresis on a 4-20% gel (Bio-Rad, CA) and transferred to 0.45 μm pore size nitrocellulose membrane (GE Healthcare Bio-Sciences, PA). Each membrane was blocked with 1X Tris-Buffered Saline and Tween 20 (TBST) (Santa Cruz Biotechnology, CA) containing 3% fat-free milk and then incubated overnight with Egr-1 (588), GADD45α (H-165), Elk-1 (H-20) and β-actin (C4) (1:200 dilution in 1X TBST plus 5% Bovine Serum Albumin (BSA) and 0.02% NaN3; Santa Cruz Biotechnology, CA), respectively. The following day, the membrane was incubated with a secondary donkey anti-rabbit IgG or goat anti-mouse IgG conjugated to peroxidase (Santa Cruz Biotechnology, CA) and diluted 1:5,000 in 1X TBST with 3% fat-free milk. Detection was carried out using the SuperSignal West Pico chemiluminesce substrate (Thermo Scientific, IL).

### Quantitative real-time polymerase chain reaction (qRT-PCR)

Total RNA was isolated from 1 × 10^6^ A549 cells treated with 100 μM of resveratrol for various hours using Column-PureTM total tissue RNA isolation kit (Lamda Biotech, MO) according to manufacturer's recommendation. Reserve transcription reactions were carried by using AccuPower® RT PreMix (Bioneers, NM) following manufacturer's protocols. Real-time PCR was carried out using Applied Biosystems® 7900HT fast Real-time PCR system (Life technologies, CA). The cDNA generated from reverse transcription was diluted 1:10 and 5μl was utilized to conduct PCR. PCR reactions were carried out in microAmp® fast optical 96-well reaction plates (Life technologies, CA) with Evagreen qPCR master mix (2X) (LAMDA Biotech, MO), forward and reverse primers (0.3 μM) (Eurofins) in a final PCR reaction volume of 20 μl. The primers for Egr-1 and the endogenous control GAPDH were the same as previously reported [17]. Amplification parameters were: denaturation at 95°C 10 min, followed by 40 cycles of 95°C, 15 s; 60°C, 60 s. Samples were analyzed in duplicate. Fold induction was calculated using the formula 2-ßßCt, where ßCt = target gene Ct − GAPDH Ct, and ßßCt is based on the mean ßCt of respective control (non-resveratrol treated).

### Simultaneous fluorescence in situ hybridization and immunofluorescence

Simultaneous fluorescence in situ hybridization (FISH) and immunofluorescence was performed following the protocol provided by Biosearch Technologies (CA). 5,000 cells were seeded on 18 mm round coverslip on day one, and treated with 100 μM of resveratrol as time indicated on day two. Medium was aspirated and cells were washed with 1X PBS and then fixed with formaldehyde (3.7% in 1X RNase free PBS) for 10 min. After the fixation buffer was removed, cells were washed with 1X PBS twice and permeabilized with 70% ethanol at 4°C overnight. On day four, the ethanol was removed and wash buffer (10% formamide in 2X saline-sodium citrate (SSC) was added to cells for 2-5 min. Then the wash buffer was aspirated and 100 μl of Egr-1 Stellaris FISH probe (1 μl from 25 μM stock solution) plus GADD45α or Elk-1 primary antibody (0.5 μg/ml as final concentration) in hybridization buffer (10% formamide in 2X SSC plus 100 μg/ml of dextran sulfate) was added on a layer of parafilm. The coverslip was placed over the hybridization buffer and incubated in a dark humidified chamber at 37°C overnight. On the following day, the coverslip was removed from hybridization buffer and incubated in 1 ml of wash buffer including 10 μg/ml of corresponding fluorescently-labeled secondary antibody again at 37°C for 30 min in the dark. One drop of mounting medium containing 1.5 μg/ml of 4′, 6-diamidino-2-phenylindole (DAPI) was used to mount cells on the slide. DAPI was used as a DNA counterstain. The cells were imaged using Axio Imager M2 from Carl Zeiss at 400X magnification. Custom Egr-1 probe were designed from Biosearch technologies, which contained a mix of multiple 20-mer oligonucleotides, each labeled with a single CAL Fluor Red 590. Egr-1 probe sets contained 37 oligos.

### Inhibitor studies

The final concentrations of all the inhibitors were 10 μM except SP 600125 (BIOMOL International, PA), of which the concentration was 20 μM. SB 203580 (Promega, WI) inhibits p38MAPK/SAP2, whereas SP 600125 is a potent JNK inhibitor. U-0126 (Santa Cruz Biotechnology, Inc, TX) is a potent inhibitor of ERK1/2 activity and LY 294002 (Santa Cruz Biotechnology, Inc, TX) blocks PI3K/AKT pathways. Cells were treated with or without 100 μM of resveratrol for another 6 h after 1 h preincubation, and the expression of Egr-1 protein and β-actin (loading control) was examined by western blotting.

### *In vitro* dual-luciferase assay

A549 cells were plated at 1 × 104 cells per well in 96-well plate and transfected with the firefly luciferase reporter constructs (pE460, pE5, pE6, pE9NS). All groups were co-transfected with the Renilla luciferase construct pRL-TK to normalize transfection efficiency. After 48 h, cells were treated with 100 μM of resveratrol overnight, and luciferase activity was measured using the Dual-Luciferase reporter assay system (Promega, WA).

### Construction of suicide gene therapy vector

GADD45α open reading frame (ORF) was PCR amplified from GADD45α cDNA (Origene, MD) using specific primers:

F- 5′-GGATCCGCCACCATGACTTTGGAGGAATTCTCG-3′

R- 5′-ATTCGCGGCCGCTCACCGTTCAGGGAGATTAAT-3′

pTarget vector was linearized with fast digest BamHI and fast digest NotI enzymes at 37°C for 10 min and linearized vector was purified by gel purification Qiagen kit (Qiagen Sciences, MD). Fusion cloning was performed with purified GADD45α cDNA and linearized pTarget using infusion dry HD cloning kit (Clontech Laboratories, Inc., CA) to create pT.G45α.

460 bp of Egr-1 promoter upstream of start site was PCR amplified on pEgr-1-luc using specific primers:

E460F- 5′-ATGGCTC GACAGATCTGCTTGGAACCAGGGAGGAG

E460R- 5′-TCAACGGGGCGGG CGATCGCGGCCTCTATTTGAAGGGTCTGG

pT.G45α vector was linearized with fast digest BgIII and fast digest AsiSI enzymes at 37°C for 15-20 min and linearized vector was purified by gel purification Qiagen kit (Qiagen Sciences, MD). Purified PCR fragment and linearized pT.G45α were mixed and added to dry pellet of infusion enzyme followed by incubation at 37°C for 15 min and 50°C for 15 min to construct pE460.G45α.

Synthetic Egr-1 enhancer sequences were prepared using the following oligonucleotides (ODN):

E9NSODN1- 5′-[Phos]GATCT(CCATATAAGG)9GCGAT-3′

E9NSODN2- 5′-[Phos]CGC(CCTTATATGG)9A-3′

Each pair of ODNs was incubated with 1X oligo annealing buffer containing 10 mM Tris-HCl pH 8.0, 100 mM NaCl, and 10 mM EDTA at 95°C for 4 min and cool at room temperature for 5-10 min to create double ODN (dODN). Ligation between dODN and linearized pT.G45α was performed by incubating with T4 ligase and T4 ligase buffer at 22°C for 1 h and then heated at 70°C for 5 min to create pE9NS.G45α.

### *In vitro* transfection

All transfections were performed using magnetofection principle. Briefly, A549 cells were seeded in various plates in Opti-MEM® containing 10% FBS and 1% NEAA without antibiotics the day before transfection. When cell confluency reached 90-95%, transfection were performed using Lipofectamine 2000 (Invitrogen, CA) and CombiMag reagent (OZ Biosciences) according manufacturer's instruction. In general, 2-3 μl of LipofectamineTM 2000 and 1 μl of CombiMag were used for the transfection of per μg of DNA or every 40 pmol of siRNA. After the complexes were added to each well, the plate was incubated on a magnetic plate for 20 min.

### Cell viability assay

A549 cells (1 × 104 per well) were seeded in 96-well plates. After transfection and resveratrol treatment, 20 μl of MTT (5 mg/ml) was added to each well and incubated for 4 h. Media was exchanged with 100 μL of DMSO and the absorbance at 570 nm was recorded.

### Apoptosis

Cells (5 × 105 per well) were seeded in 6-well plates and transfected with vector pE9NS.G45α or pT.Luc for 24 h followed by 100 μM of resveratrol treatment for additional 24 h. Cells were harvested and stained with Annexin V-FITC and propidium iodide (PI) using Apoptosis Detection Kit (Life Technologies, CA). Flow cytometry was performed using BD AccuriTM C6 and the data were analyzed by CFlow Plus (BD Biosciences, NJ).

## References

[1] Jin S, Tong T, Fan W, Fan F, Antinore MJ, Zhu X, Mazzacurati L, Li X, Petrik KL, Rajasekaran B, Wu M, Zhan Q (2002). GADD45-induced cell cycle G2-M arrest associates with altered subcellular distribution of cyclin B1 and is independent of p38 kinase activity. Oncogene.

[2] Ettinger DS, Akerley W, Bepler G, Blum MG, Chang A, Cheney RT, Chirieac LR, D'Amico TA, Demmy TL, Ganti AK, Govindan R, Grannis FW, Jahan T (2010). Non-small cell lung cancer. J Natl Compr Canc Netw.

[3] Salvador JM, Brown-Clay JD, Fornace AJ (2013). Gadd45 in stress signaling, cell cycle control, and apoptosis. Adv Exp Med Biol.

[4] Greco O, Powell TM, Marples B, Joiner MC, Scott SD (2005). Gene therapy vectors containing CArG elements from the Egr1 gene are activated by neutron irradiation, cisplatin and doxorubicin. Cancer Gene Ther.

[5] Zhan Q, Antinore MJ, Wang XW, Carrier F, Smith ML, Harris CC, Fornace AJ (1999). Association with Cdc2 and inhibition of Cdc2/Cyclin B1 kinase activity by the p53-regulated protein Gadd45. Oncogene.

[6] Takekawa M, Saito H (1998). A family of stress-inducible GADD45-like proteins mediate activation of the stress-responsive MTK1/MEKK4 MAPKKK. Cell.

[7] Yang F, Zhang W, Li D, Zhan Q (2013). Gadd45a suppresses tumor angiogenesis via inhibition of the mTOR/STAT3 protein pathway. J Biol Chem.

[8] Higashi H, Vallbohmer D, Warnecke-Eberz U, Hokita S, Xi H, Brabender J, Metzger R, Baldus SE, Natsuqoe S, Aikou T, Holscher AH, Schneider PM (2006). Down-regulation of Gadd45 expression is associated with tumor differentiation in non-small cell lung cancer. Anticancer Res.

[9] Al-Romaih K, Sadikovic B, Yoshimoto M, Wang Y, Zielenska M, Squire JA (2008). Decitabine-induced demethylation of 5′ CpG island in GADD45A leads to apoptosis in osteosarcoma cells. Neoplasia.

[10] Zerbini LF, Libermann TA (2005). GADD45 deregulation in cancer: frequently methylated tumor suppressors and potential therapeutic targets. Clin Cancer Res.

[11] Sheikh MS, Hollander MC, Fornance AJ (2000). Role of Gadd45 in apoptosis. Biochem Pharmacol.

[12] Tong T, Ji J, Jin S, Li X, Fan W, Song Y, Wang M, Liu Z, Wu M, Zhan Q (2005). Gadd45a expression induces Bim dissociation from the cytoskeleton and translocation to mitochondria. Mol Cell Biol.

[13] Cao XM, Koski RA, Gashler A, McKiernan M, Morris CF, Gaffney R, Hay RV, Sukhatme VP (1990). Identification and characterization of the Egr-1 gene product, a DNA-binding zinc finger protein induced by differentiation and growth signals. Mol Cell Biol.

[14] Sukhatme VP, Cao XM, Chang LC, Tsai-Morris CH, Stamenkovich D, Ferreira PC, Cohen DR, Edwards SA, Shows TB, Curran T, Beau MM, Adamson ED (1988). A zinc finger-encoding gene coregulated with c-fos during growth and differentiation, and after cellular depolarization. Cell.

[15] Datta R, Rubin E, Sukhatme V, Qureshi S, Hallahan D, Weichselbaum RR, Kufe DW (1992). Ionizing radiation activates transcription of the EGR1 gene via CArG elements. Proc Natl Acad Sci U S A.

[16] Khachigian LM, Anderson KR, Halnon NJ, Gimbrone MA, Resnick N, Collins T (1997). Egr-1 is activated in endothelial cells exposed to fluid shear stress and interacts with a novel shear-stress-response element in the PDGF A-chain promoter. Arterioscler Thromb Vasc Biol.

[17] Shi Q, Sutariya V, Bishayee A, Bhatia D (2014). Sequential activation of Elk-1/Egr-1/GADD45ɑ by arsenic. Oncotarget.

[18] Joki T, Nakamura M, Ohno T (1995). Activation of the radiosensitive EGR-1 promoter induces expression of the herpes simplex virus thymidine kinase gene and sensitivity of human glioma cells to ganciclovir. Hum Gene Ther.

[19] Lopez CA, Kimchi ET, Mauceri HJ, Park JO, Mehta N, Murphy KT, Beckett MA, Hellman S, Posner MC, Kufe DW, Weichselbaum RR (2004). Mol Cancer Ther.

[20] Scott SD, Joiner MC, Marples B (2002). Optimizing radiation-responsive gene promoters for radiogenetic cancer therapy. Gene Ther.

[21] Marple B, Greco O, Joiner MC, Scott SD (2002). Molecular approaches to chemo-radiotherapy. Eur J Cancer.

[22] Weichselbaum RR, Hallahan DE, Beckett MA, Mauceri HJ, Lee H, Sukhatme VP, Kufe DW (1994). Gene therapy targeted by radiation preferentially radiosensitizes tumor cells. Cancer Res.

[23] Athar M, Back JH, Kopelovich L, Bickers DR, Kim AL (2009). Multiple molecular targets of resveratrol: anti-carcinogenic mechanisms. Arch Biochem Biophys.

[24] Signorelli P, Ghidoni R (2005). Resveratrol as an anticancer nutrient: molecular basis, open questions and promises. J Nutr Biochem.

[25] Bickenbach KA, Veerapong J, Shao MY, Mauceri HJ, Posner MC, Kron SJ, Weichselbaum RR (2008). Resveratrol is an effective inducer of CArG-driven TNF-ɑ gene therapy. Cancer Gene Therapy.

[26] Marples B, Greco O, Joiner MC, Scott SD (2002). Molecular approaches to chemo-radiotherapy. Eur J Cancer.

[27] Whitlock NC, Bahn JH, Lee SH, Eling TE, Baek SJ (2011). Resveratrol-induced apoptosis is mediated by early growth response-1, Krüppel-like factor 4, and activating transcription factor 3. Cancer Prev Res (Phila).

[28] Whitmarsh AJ, Shore P, Sharrocks AD, Davis RJ (1995). Integration of MAP kinase signal transduction pathways at the serum response element. Science.

[29] Shih A, Davis FB, Lin HY, Davis PJ (2002). Resveratrol induces apoptosis in thyroid cancer cell lines via a MAPK- and p53-dependent mechanism. J Clin Endocrinol Metab.

[30] She QB, Bode AM, Ma WY, Chen NY, Dong Z (2001). Resveratrol-induced activation of p53 and apoptosis is mediated by extracellular-signal-regulated protein kinases and p38 kinase. Cancer Res.

[31] Gregg J, Fraizer G (2011). Transcriptional regulation of EGR1 by EGF and the ERK signaling pathway in prostate cancer cells. Genes Cancer.

[32] Thyss R, Virolle V, Imbert V, Peyron JF, Aberdam D, Virolle T (2005). NF-kappaB/Egr-1/Gadd45 are sequentially activated upon UVB irradiation to mediate epidermal cell death. EMBO J.

[33] Quiñones A, Dobberstein KU, Rainov NG (2003). The egr-1 gene is induced by DNA-damaging agents and non-genotoxic drugs in both normal and neoplastic human cells. Life Sci.

[34] Shaulian E, Karin M (1999). Stress-induced JNK activation is independent of Gadd45 induction. J Biol Chem.

[35] Hollander MC, Sheikh MS, Bulavin DV, Lundgren K, Augeri-Henmueller L, Shehee R, Molinaro TA, Kim KE, Tolosa E, Ashwell JD, Rosenberg MP, Zhan Q, Fernández-Salguero PM (1999). Genomic instability in Gadd45a-deficient mice. Nat Genet.

[36] Hollander MC, Kovalsky O, Salvador JM, Kim KE, Patterson AD, Haines DC, Fornace AJ (2001). Dimethylbenzanthracene carcinogenesis in Gadd45a-null mice is associated with decreased DNA repair andincreased mutation frequency. Cancer Res.

[37] Li Y, Qian H, Li X, Wang H, Yu J, Liu Y, Zhang X, Liang X, Fu M, Zhan Q, Lin C (2009). Adenoviral-mediated gene transfer of Gadd45a results in suppression by inducing apoptosis and cell cycle arrest in pancreatic cancer cell. J Gene Med.

[38] Cottart CH, Nivet-Antoine V, Laguillier-Morizot C, Beaudeux JL (2010). Resveratrol bioavailability and toxicity in humans. Mol Nutr Food Res.

[39] Ragione FD, Cucciolla V, Criniti V, Indaco S, Borriello A, Zappia V (2003). p21Cip1 gene expression is modulated by Egr1: a novel regulatory mechanism involved in the resveratrol antiproliferative effect. J Biol Chem.

[40] Li G, He S, Chang L, Lu H, Zhang H, Zhang H, Chiu J (2011). GADD45ɑ and annexin A1 are involved in the apoptosis of HL-60 induced by resveratrol. Phytomedicine.

[41] Weichselbaum RR, Kufe D (2009). Translation of the radio- and chemo-inducible TNFerade vector to the treatment of human cancers. Cancer Gene Ther.

[42] Hsu H, Rainov NG, Quinones A, Eling DJ, Sakamoto KM, Spear MA (2003). Combined radiation and cytochrome CYP4B1/4-ipomeanol gene therapy using the EGR1 promoter. Anticancer Res.

